# Comparison of early and late intravenous gamma globulin treatment of Kawasaki disease on fever and cardiovascular complications

**Published:** 2016

**Authors:** Iraj Mohammadzadeh, Somayyeh Noei, Kazem Babazadeh, Hassan Zamani, Rahim Barari-Savadkoohi, Reza Alizadeh-Navaei

**Affiliations:** 1 Non-Communicable Pediatric Research Center, Babol University of Medical Sciences, Babol, Iran.; 2 Infectious Diseases and Tropical Medicine Research Center, Babol University of Medical Sciences, Babol, Iran.; 3 Gastrointestinal Cancer Research Center, Mazandaran University of Medical Sciences, Sari, Iran.

**Keywords:** Kawasaki, Cardiac complications, Children, IVIG

## Abstract

**Background:**

Cardiac involvement was the major leading cause of death in patients with Kawasaki and IVIG administration reduces cardiac complications. The objective of this study was to determine the frequency of cardiovascular complications and duration of fever with regard to the time of intravenous immunoglobulin (IVIG) administration of patients with Kawasaki disease.

**Methods:**

This follow-up study was done on all patients with Kawasaki disease who were hospitalized at Amirkola Children’s Hospital between 2006 and 2011. Diagnosis of Kawasaki was clinical and included fever more than 5 days with 4 of 5 signs containing mucosal changes, scaling and skin rash, bilateral nonexudative conjunctivitis, cervical lymph adenopathy and edema in lower extremities. After diagnosis of Kawasaki, all patients received standard treatment (intravenous immunoglobulins and aspirin) and undergoing cardiac echocardiography in 2 weeks, 2 months and 6 months. Information including age, sex, sign of diseases, laboratory findings, and cardiac complications in echocardiography were recorded.

**Results:**

This study was performed on 100 patients (61 boys and 39 girls) with Kawasaki disease. The mean age of children was 2.8±2.6 years. Cardiac complication rate was 47% at the onset of the disease and had reached to 7% at the end of the sixth month (P=0.000). Distribution of cardiovascular complications in the second week, the second month and the sixth month after treatment was not significantly different according to the start of time of treatment (p>0.05). Duration of fever in patients who received treatment before 10^th^ day (1.5±1.3) did not have significant difference (P=0.78) with patients who received after 10^th^ day (1.6±0.9).

**Conclusion:**

Result shows that most of patients (99%) responded to the treatment with IVIG and ASA and cardiovascular complication ratio decreased. There was not significant relationship between duration of fever and time of IVIG treatment initiation.

Kawasaki disease (KD) is an acute febrile vasculitis that is usually observed in neonates and children (1). The disease was first described by Kawasaki in 1967. Currently, the disease is one of the causes of acquired heart diseases including coronary artery diseases in children (2). In the study performed by Saffar et al., the annual disease prevalence was reported to be 7.3 per 100000 people (3). In the latest studies, it has been shown that the disease incidence is increasing in recent years (2, 4, 5).

Major symptoms of the disease include fever, bilateral conjunctival injection without exudate, erythema of lips and oral mucosa, acral changes, rash, and cervical lymphadenopathy (6). Among the patients remained untreated, 20-25% may experience coronary artery abnormalities, aneurysm rupture, and sudden death (7). Moradinezhad and Kiani reported the prevalence of coronary artery aneurysm in KD patients as 38% in the study performed in Tehran (8). The KD patients are treated by intravenous immunoglobulin (IVIG) and high-dose oral aspirin (2). Early diagnosis and treatment of the disease would lead to reduced complications, particularly the cardiovascular complications. Considering this and the possible effect of environmental factors, the current study was performed to determine the frequency of cardiovascular complications and duration of fever with regard to the time of IVIG administration in KD patients among all the patients hospitalized with the diagnosis of KD.

## Methods

This follow-up study includes all KD patients who were hospitalized in the Amirkola Children’s Hospital during 2006-2011. Diagnosis of KD was clinical and based upon the presence of fever persisted for more than five days accompanied by at least four items of the followings; mucosal changes, skin rashes and desquamation, bilateral conjunctival injection without exudate, cervical adenopathy, and acral edema and excluding other febrile diseases (9). Patients who did not meet full criteria were considered as incomplete KD in terms of echocardiographic results.

After diagnosis, all patients were treated by IVIg. The treatment used for all patients was the standard treatment of single-dose IVIg (2 g/kg) over 10-12 hours and aspirin (100 mg/kg/day in four divided doses) for two weeks and then 5 mg/kg/day for two months. All patients underwent echocardiography two weeks, two months, and then six months later. Echocardiography study was performed using Medison Accuvix V10 with two probes.

For each patient, data regarding age, sex, presence and duration of fever, development of complications over the study period and laboratory test results were recorded in checklists. White blood cell (WBC) count and CRP levels were classified according to the normal values for different age ranges. Thrombocytosis was considered as the platelet count above 450000/µl, and sterile pyuria was defined as the presence of more than five WBCs in the urinalysis (7). ESR level was classified as above 50 (high) and below 50 (8). Since initiation of the treatment within 7-10 days after onset of the fever is accompanied by the lowest rate of cardiovascular complications (10), we categorized the initiation of treatment up to 10 days and after 10 days of the fever onset. Aneurysm was considered as the coronary artery internal luminal diameter more than 3 mm in children less than 5 years old and more than 4 mm in children above 5 years old or diameter of involved coronary artery >1.5 times greater than adjacent normal artery.

Ectasia defined as the diameter of the aneurysm was more than 1.5 times the size of the normal diameter, but internal luminal diameter was less than 3 mm in children less than 5 years old and less than 4 mm in children above 5 years old. Brightness was defined as the normal size together with inflammation of the coronary artery wall (9).Data were analyzed using the SPSS software, Version 17 by the t-test, Fisher’s exact test, and Cochran’s test. P-values less than 0.05 were considered statistically significant.

## Results

The study was performed on 100 KD patients, including 61 boys. The mean age of patients was 2.8±2.6 years (range: 4 month – 15 year). Duration of fever was 9.7±4 days (in the range of 5-26 days). Distribution of other symptoms are presented in table 1. 

**Table 1:** Frequency distribution of KD symptoms in the study participants (n=100

**Symptom **	**Frequency or Percentage**	**Symptom**	**Frequency or Percentage**
Conjunctival injection without exudate	70	Swelling dorsa of the hands	11
Redness and cracking of lips	83	Swelling dorsa of the feet	14
Strawberry tongue	50	Cervical lymphadenopathy	41
Oropharynx redness	77	Skin rashes	74
Erythema of palms	19	Acral desquamation	9
Erythema of soles	14	Perianal desquamation	7

As shown in table 1, the most prevalent symptom was fever (100%) followed by redness and cracking of lips, oropharynx redness, skin rashes, and conjunctival injection without exudate. Among the patients, 23 had incomplete KD, while 77 had typical KD. However, aneurysm was observed in 15 patients (at the beginning and during the study). Distribution of the laboratory findings are given in table 2.

**Table 2:** Distribution of the laboratory findings in KD study participants

**Laboratory findings **	**Frequency (%)**
Leukocytosis	66 (66)
Thrombocytosis	73 (73)
Sterile pyuria *	23 (24.5)
High CRP level **	82 (92.1)
High ESR level ***	94 (94.9)

* 6 missing cases

** 11 missing cases

*** 1 missing case

In 65 patients, the treatment was initiated within 10 days after fever onset, while in 35 patients, treatment started 10 days after fever onset. Duration of fever after initiation of the treatment was 1.5±1.2 days range, 1-8 days). Since the fever continued in nine patients (9%), they received the second dose of IVIG, among which six had typical KD and three had incomplete KD. Moreover, among these nine patients, fever continued in one patient despite receiving two doses of IVIg, and thus, the pulse therapy of methyl prednisolone (30mg/Kg/day) was applied. The primary rests of echocardiography are presented in table 3, 4. As it is observed, 53 patients had normal echocardiography. As shown in figure 1, frequency of cardiovascular complications decreased from 47% at the disease onset to 13% at the end of the sixth month.

As given in table 4, distribution of cardiovascular complications in 2nd week, 2nd month, and 6th month after beginning of treatment was not significantly different with regard to the different times of treatment initiation. Duration of fever in patients who received the treatment up to day 10 was 1.5±1.3 days. The duration was 1.6±0.9 days in those that received the treatment after day 10. The two groups were not significantly different in this respect (p= 0.78).

As shown in table 5, distribution of cardiovascular complications in the 2nd week, the 2nd month, and the 6th month after treatment initiation was not significantly different considering the disease type.

**Table 3:** Frequency distribution of primary echocardiography findings in the KD patients studied at different times

**Echocardiography finding **	**Time**			
	**Basal**	**2nd week**	**2nd month**	**6th month**
Aneurysm	7	12	14	12
Ectasia	27	30	8	1
Brightness	18	2	9	0
Myocarditis / pericarditis.	7	4	2	1
Tricospid regurgitation	2	1	0	0
Mitral regurgitation	1	1	0	0
Normal	53	58	80	87

**Table 4:** Frequency distribution and percentage of cardiovascular complications with regard to the time of treatment initiation in KD patients studied

Time of treatment initiation Time and result of echocardiography	10 days after fever onset N (%)	After 10 days after fever onset N (%)	P value
2nd week			
Normal Complicated Total	39 (60) 26 (40) 65 (100)	19 (54.3) 16 (45.7) 35 (100)	0.627
2nd month			
Normal Complicated Total	53 (81.5) 12 (18.5) 65 (100)	27 (77.1) 8 (22.9) 35 (100)	0.609

**Diagram 1:**
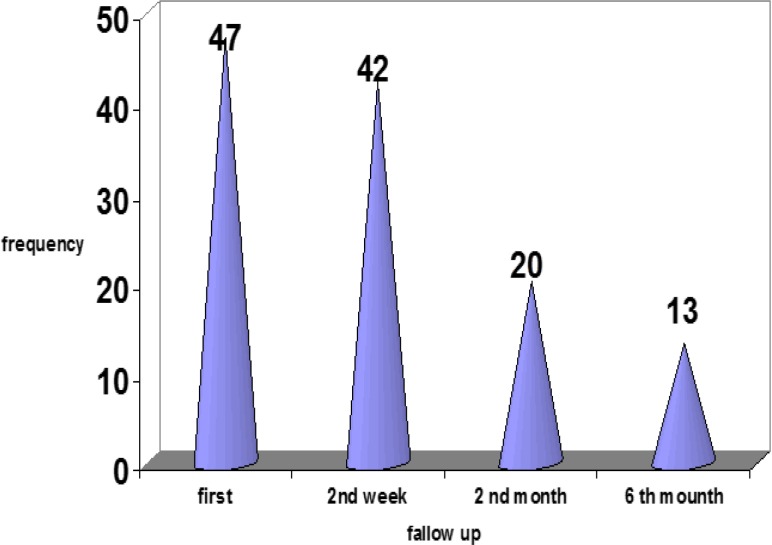
Frequency distribution of cardiovascular complications at different times in the KD patients studied (P= 0.000).

**Table 5:** Frequency distribution and percentage of cardiovascular complications with regard to the type of KD in patients studied

KD type Time and result of echocardiography	Typical N (%)	Typical N (%)	P value
2nd week			
Normal Complicated Total	45 (60) 32 (40) 77 (100)	45 (60) 32 (40) 77 (100)	1
2nd month			
Normal Complicated Total	60 (77.9) 17 (22.1) 77 (100)	60 (77.9) 17 (22.1) 77 (100)	0.553
6th month			
Normal Complicated Total	67 (87) 10 (13) 77 (100)	67 (87) 10 (13) 77 (100)	1

## Discussion

Kawasaki disease is a systemic vasculitis that most commonly affect children under 5. Major complications of the disease are cardiovascular complications. Early administration of IVIG (within first 10 days of the disease) would reduce the risk of cardiovascular complications. The present study was performed to determine the frequency of fever and cardiovascular complications following IVIG administration in KD patients. 

Our results showed that the disease frequency is higher in males, with the male-to-female ratio of 1.5:1. In the study carried out by Gil Veloz et al. in Mexico, this ratio was 1.4:1 (11). In Sharif and Iranfar’s study in Kashan and Tehran, among the 63 patients with KD, 40 were males (12). Accordingly, in Ayazi et al.’s study. On 20 treated KD patients, the male-to-female ratio was 1:0.45 (13). Kordidarian et al. performed a study on 45 KD patients in Isfahan, and reported the male-to-female ratio as 1.8:1 (14). However, Morales-Quispe et al. performed a study in Mexico and found the male-to-female ratio as 1:1.2 (15). In this respect, the current study is similar to the studies mentioned above. Only in the Morales-Quispe et al.’s study, the disease frequency was higher in females, which may be attributed to the small sample size, as they performed the study only on 11 patients. In the present study, more than half of the patients were under two years old and 91% were under six. In the Morales-Quispe et al.’s study, 80% of the KD patients were under 5 (15). Gil Veloz et al. reported that 82% of KD patients were under 5 (11). As it was expected, most affected children were at early years of life and the disease frequency decreased with increasing age.

In the current study, the most prevalent symptom was fever (100%) followed by redness and cracking of lips (83%), oropharynx redness (77%), skin rashes (74%), and conjunctival injection without exudate (70%). Morales-Quispe et al. reported eye redness as the commonest clinical presentation, which was present in 80% of the cases (15). In the study performed by Gheini et al. in Kermanshah, fever was observed in all patients, while acral changes, bilateral conjunctival injection, and upper respiratory tract changes were observed in 95.6%, 91.3%, and 86.9% of the patients, respectively (16). 

Furthermore, in the study performed by Mahmoudzadeh et al. fever was observed in all patients, followed by skin rashes (93%), marked cervical lymphadenopathy (64%), acral changes (62%), bilateral conjunctival injection without exudate (73%), and lip and mouth mucosal changes (86%) (17). In the present study and also other studies, it is shown that the disease symptoms are present in many patients. This emphasizes the importance of paying attention to accompanying symptoms of children referred by prolonged fever.

The laboratory findings in the study were leukocytosis (66%), thrombocytosis (73%), sterile pyuria (24.5%), high levels of CRP (92.1%), and high levels of ESR (94.9%). Sharif and Iranfar reported the laboratory findings as high ESR levels (100%), thrombocytosis (90%), leukocytosis (82.5%), and proteinuria and pyuria (53.9%) (12). In the study carried out by Rahbari-manesh et al., ESR levels above 90, positive CRP, and platelet count above 450000 were observed in 71.4%, 92.8%, and 78.6% of the patients, respectively (18). Common laboratory findings in the study performed by Ayazi et al. were high ESR levels (75.9%), positive CRP (62.1%), thrombocytosis (44.8%), and leukocytosis (37.9%) (13). In the study carried out by Kordidarian et al., high ESR levels, positive CRP, thrombocytosis, sterile pyuria, leukocytosis, and proteinuria were observed in 93.3%, 77.5%, 74.3%, 53.7%, 53.3%, and 34.2% of the patients, respectively (14). In the study performed by Mosaiebi et al. in Kashan, 89.9% of the patients had high levels of ESR (19). Considering the results mentioned, it is concluded that high levels of ESR and CRP are observed in most patients.

In the current study, the rate of cardiovascular complications reached from 47% at the disease onset to 13% at the end of the sixth month. This indicates that administration of IVIG would significantly reduce the rate of cardiovascular complications in KD patients and the rates of coronary complication among early and late IVIG treatment did not have significant difference. In the study performed by Sano et al. in Japan, the rate of cardiovascular complications in the patients that received IVIG was 5%, while the rate was 71% among the non-responsive patients (20). In the study performed by Du et al. in China, the incidence rate of coronary artery complications in the group that received conventional treatment was 18.3% (21).

In our study, duration of fever in the patients for whom the treatment was initiated up to day 10 was less than that in patients who received the treatment after day 10. However, the difference was not statistically significant (p>0.05). Du et al. reported that the rate of IVIG non-responders was higher in early as compared with conventional and late treatment group (21). However, either clinically in our study or statistically in the studyperformed by Du et al., the administration of IVIG would lead to earlier resolution of fever.

In our study, distribution of cardiovascular complications in the 2nd week, the 2nd month, and the 6th month after treatment initiation was not significantly different with regard to the different times of the treatment initiation. However, the rate of complications was lower in patients who received the treatment earlier. Tse et al. performed a study in Canada and found that three months after the disease onset, the rate of cardiovascular complications was not significantly different in the group that received IVIg earlier compared with the group that received the treatment later (22). Our results are consistent with this study. Nevertheless, Du et al. reported that the rate of coronary artery complications in the groups that received conventional and late treatments were 18.3% and 33.7%, respectively (21). Also, Kordidarian et al. showed that the frequency of coronary artery aneurysm among the patients that were diagnosed and received IVIG within 10 days of their referral was lower than those in patients who were referred after 10 days of the disease onset (14). To compare the results of these studies indicating that earlier administration of IVIG would at least clinically reduce the incidence rate of cardiovascular complications among KD patients.

**Conclusion: **The results obtained indicate that most KD patients responded to IVIG (99%) and aspirin administration appropriately and the treatment reduced the rate of cardiovascular complications. However, a statistically significant relationship was not observed between duration of the disease complications and time of treatment initiation.

## Notes

**Funding: **This project was supported by Babol University of Medical Sciences.

## Citation:

Mohammadzadeh I, Noei S, Babazadeh K, et al. Comparison of early and late intravenous gamma globulin treatment of Kawasaki disease on fever and cardiovascular complications. Caspian J Intern Med 2016; 7(3): 211-216.
